# Wild birds as environmental reservoirs of antimicrobial-resistant *Salmonella*: a global systematic review and meta-analysis

**DOI:** 10.3389/fmicb.2026.1787396

**Published:** 2026-04-24

**Authors:** Eurade Ntakiyisumba, Waruni Ekanayake, Maryum Tanveer, Fabrice Hirwa, Noutlady Inthavong, Eric Sibomana, Gayeon Won

**Affiliations:** Bio-Safety Research Institute and College of Veterinary Medicine, Jeonbuk National University, Iksan, Republic of Korea

**Keywords:** antimicrobial resistance, meta-analysis, prevalence, *Salmonella*, wild birds, wildlife

## Abstract

**Background:**

The lack of consolidated global data on the prevalence and antimicrobial resistance (AMR) profiles of *Salmonella* in wild bird populations hinders our understanding of their epidemiological role as reservoirs and disseminators of resistant strains and impedes accurate evaluation of their potential implication for global public health.

**Methods:**

A random-effects meta-analysis was conducted to generate a pooled prevalence estimate of *Salmonella* and its AMR patterns in the global wild bird populations.

**Results:**

The pooled prevalence of *Salmonella* spp. in wild birds was 5.77% (95% CI: 4.21–7.54%), with the highest prevalence observed in Asia (10.13%), followed by Africa (6.66%) and Europe (6.14%). Serovar-specific analysis revealed that *S*. Typhimurium had the highest prevalence (4.12%), followed by *S*. Enteritidis (1.42%). Significant variation in prevalence across avian taxonomic orders has been detected, with Accipitriformes (6.85%) and Charadriiformes (6.15%) exhibiting the highest infection rates. Resistance to critically important antimicrobials ranged from 0% to 29.6%, with the highest prevalence observed for macrolides (29.6%), monobactams (22.9%), and penicillins (14.7%). In addition, a significant temporal increase in resistance was observed for key antimicrobials, including fluoroquinolones (*R*^2^ = 19.21%, *p* = 0.003*)* and penicillin/β*-*lactamase inhibitors (*R*^2^ = 66.75%, *p* < 0.01), suggesting an escalating environmental selective pressure.

**Conclusions:**

A significant temporal increase to some critically important antimicrobials highlights the growing influence of environmental selective pressures on AMR dynamics in wild bird populations. These trends suggest increasing environmental exposure to antimicrobial residues and resistant determinants, reinforcing the role of wildlife as sentinels and potential reservoirs for clinically relevant resistance with important implications for ecosystem health and public health surveillance. Given the interconnectedness between wildlife, livestock, and humans, the presence of AMR *Salmonella* isolates in wild birds represents a potential public health concern, even when resistance levels are low to moderate. These findings support the need for integrated One Health surveillance approaches and coordinated global policy interventions to monitor and mitigate the dissemination of resistance across ecosystems.

## 1 Introduction

Wild birds have been identified as important reservoirs and potential vectors of foodborne bacteria such as *Salmonella* spp., thus posing considerable threats to public health, animal welfare, and environmental stability (Wiederkehr et al., 2024; Kobuszewska and Wysok, 2024). Their ecological diversity, migratory behavior, and frequent interaction with anthropogenically influenced environments place them at the human-livestock-environment interface, where they may contribute to the circulation of zoonotic bacteria. Numerous studies have documented the presence of *Salmonella* in wild bird populations worldwide; however, reported prevalence estimates vary widely across bird species, ecological settings, and geographic regions (Aung et al., 2019; Card et al., 2023; Marin et al., 2018). While this body of evidence clearly demonstrates that *Salmonella* incidence in wild birds is widespread, the overall magnitude of this burden remains difficult to interpret due to the absence of consolidated, quantitative prevalence estimates that integrate findings across diverse contexts.

Antimicrobial resistance (AMR) further amplifies the public health significance of *Salmonella* in wild bird populations. Although wild birds are not directly exposed to antibiotics, increasing evidence indicates that some of the *Salmonella* strains harbored in wild bird populations are resistant to multiple clinically important antibiotics, thereby contributing to the global AMR crisis (Kobuszewska and Wysok, 2024; Bonnedahl and Järhult, 2014). Such resistance is primarily acquired through exposure to human-impacted environments, including contaminated water bodies, sewage systems, agricultural waste, and landfill sites (Zeballos-Gross et al., 2021; Greig et al., 2015). However, current knowledge of AMR *Salmonella* in wild birds is largely derived from isolated, site-specific investigations, and no quantitative synthesis has yet established pooled prevalence estimates or the global distribution of resistance profiles. Consequently, the epidemiological relevance of wild birds as reservoirs and disseminators of AMR *Salmonella* remains poorly quantified.

Several studies have revealed epidemiological links between AMR *Salmonella* strains detected in wild birds and those circulating in livestock and human populations, indicating potential cross-species transmission and shared environmental reservoirs of resistance determinants (Marin et al., 2018; Blanco, 2018). Such cross-species transmission may occur through both spillover, defined as the transmission of pathogens from a primary reservoir host to a novel host species, and spillback, whereby newly infected host populations become secondary reservoirs capable of retransmitting pathogens to the original host populations or other susceptible hosts (Shapiro et al., 2021). These transmission processes are particularly relevant at the human-livestock-environment interface, where ecological interactions facilitate the circulation of antimicrobial-resistant bacteria. In particular, wild birds that exploit anthropogenic food sources or migrate across long distances may act both as sentinels of environmental contamination and as carriers of antimicrobial-resistant bacteria across ecosystems and geographic boundaries (Marin et al., 2018; Bonnedahl and Järhult, 2014; Espunyes et al., 2022; Hird et al., 2015; Chen et al., 2024). These characteristics underscore the importance of wild birds within a One Health framework for understanding the environmental dimensions of AMR transmission.

Despite the growing number of studies reporting *Salmonella* and antimicrobial resistance in wild birds, the available evidence remains highly fragmented. Most investigations are limited to specific locations or host species and differ substantially in sampling strategies, sample types, laboratory methods, and antimicrobial susceptibility testing protocols. Thus, reported prevalence estimates are highly heterogeneous and difficult to compare across studies (Kang et al., 2024). Importantly, documenting the occurrence of *Salmonella* alone does not provide quantitative insight into the burden, distribution, or public health relevance of antimicrobial resistance within wild bird populations. Additionally, the absence of global pooled prevalence estimates of AMR *Salmonella* in wild birds hinders the development of coordinated international AMR surveillance frameworks and undermines the design of targeted, evidence-based public health interventions. This lack of quantitative estimates represents a significant knowledge gap that limits the integration of wildlife reservoirs into global AMR surveillance systems, risk assessments, and policy frameworks. Meta-analysis offers a robust methodological approach to address this gap by systematically synthesizing evidence from multiple studies to generate a more precise and statistically powerful pooled estimate (Paudyal et al., 2018; Field and Gillett, 2010). Its principal advantage lies in the aggregation of data from independent investigations on a particular subject, resulting in an estimate that is more robust and statistically informative than that of any single study (Paudyal et al., 2018). This approach not only improves the reliability of the research findings but also facilitates the identification and interpretation of heterogeneity across studies (Lean et al., 2009).

Herein, we conducted a systematic review and meta-analysis to estimate the global prevalence of antibiotic-resistant *Salmonella* serovars in wild bird populations. In addition, subgroup analyses were performed to explore potential sources of heterogeneity in prevalence estimates according to geographic region (i.e., country and continent), sample source, sample type, bird ecology, and antimicrobial susceptibility testing methods. By providing consolidated epidemiological evidence, this study aims to clarify the role of wild birds in the dissemination of antimicrobial-resistant *Salmonella* and to support One Health-oriented surveillance and intervention strategies.

## 2 Materials and methods

### 2.1 Study design and literature search strategy

This study investigates the prevalence of *Salmonella* spp. and the patterns of AMR in global wild bird populations through a systematic review and meta-analysis, conducted in accordance with the Preferred Reporting Items for Systematic Review and Meta-Analysis Protocols (PRISMA-P) guidelines (Page et al., 2021). The PRISMA-P guideline checklist for this study is provided in Supplementary file 1. The research question followed the “Population, Exposure, Comparator, and Outcome” (PECO) framework (Morgan et al., 2018). The “population” of interest comprised wild birds of any species, “exposure” referred to the *Salmonella* pathogen, and the “outcomes” of interest were the prevalence of *Salmonella* and/or the presence of antimicrobial-resistant *Salmonella* serovars isolated from wild bird samples. As this was a prevalence study, the “comparator” component of the PECO framework was not relevant.

An extensive literature search was conducted using electronic databases, including PubMed, Scopus, and Web of Science, to identify relevant studies published between 2000 and 2025, with no language restrictions. The final literature search was conducted on February 14, 2025. The search strategy employed the following keywords: (prevalence OR proportion OR incidence OR cases OR antimicrobial OR antibiotic OR resistan^*^ OR drug OR susceptib^*^) AND (*Salmonella* OR Salmonellosis) AND (wild bird OR migratory bird OR water bird OR bird). In addition, supplementary searches were conducted in Google Scholar to identify potentially relevant gray literature, including government reports, institutional documents, and theses. The reference lists of all eligible articles and relevant review papers were also screened using a backward snowballing approach to identify additional potentially relevant studies. All retrieved records were imported and organized using EndNote 20 (Clarivate Analytics) reference management software for further screening.

### 2.2 Eligibility and exclusion criteria

To validate the pertinence of the articles for inclusion in the meta-analysis, two independent reviewers conducted a thorough assessment of the eligibility of the retrieved articles using a set of predefined inclusion and exclusion parameters (Swift and Wampold, 2018). The inclusion criteria were as follows: cross-sectional studies, primary studies assessing the prevalence and/or AMR of *Salmonella* spp. in wild birds, studies published between 2000 and 2025, and studies reporting the sample size used and the number of positive samples. In addition, wild birds originating from rescue and rehabilitation facilities, hospitals and diagnostic centers, research centers, and conservation centers were included only when sampling was conducted immediately upon admission, prior to antimicrobial treatment or prolonged exposure to captive conditions. Therefore, these samples were considered representative of the birds' environmental exposure before entry into captive care. Studies were excluded if they were published before 2000, samples were not collected from wild birds, *Salmonella* was not the target pathogen, relevant data on the prevalence and/or AMR of *Salmonella* isolates were not available, or the full tests were not available. The screening process followed a rigorous, systematic approach. After removing duplicate records, the titles and abstracts were carefully assessed, and any potentially relevant articles were subjected to full-text review. Non-English publications were translated using Google translate to ensure their inclusion. Studies that met the predefined eligibility criteria were incorporated into both the systematic review and meta-analysis. Any discrepancies during the screening were resolved through consensus and discussion.

### 2.3 Data extraction

Two independent reviewers extracted data on the prevalence and AMR of *Salmonella* spp. from the global wild bird populations. Data on the authors, year of publication, sampling period, country, bird species, sample type, sample source, detection method, sample size, number of positive samples, total number of isolates from positive samples, number of *Salmonella*-resistant isolates, antibiotics tested, antibiotic class, and susceptibility testing method were retrieved. The extracted data were then compiled into a pre-structured Microsoft Excel spreadsheet (Schmidt et al., 2021).

### 2.4 Assessment of the risk of bias (RoB)

The methodological rigor of the eligible studies, specifically their internal and external validity, was independently evaluated by two reviewers using the Joanna Briggs Institute (JBI) critical appraisal tools for prevalence studies (Porritt et al., 2014). The application of the JBI tools facilitated a comprehensive evaluation of any potential biases and generalizability, thereby enhancing the validity of the results. The checklist consisted of nine questions, of which only eight were used to assess the studies because the question concerning response rate was deemed irrelevant to this particular investigation. The evaluated risk-of-bias components included the appropriateness of the sampling frame, study participants, sample size, description of the study subjects and settings, coverage of samples in data analysis, methods used for the identification of the condition, reliability of the methods used to measure the condition, and the appropriateness of the statistical analysis. Each study was categorized as having either a low, high, or unclear risk of bias for each bias item.

### 2.5 Meta-analysis

The meta-analysis was conducted using R statistical software (version 4.1.2) and RStudio (version 2024.12.1 + 563), using the packages “metaphor” (version 3.0.2), “meta” (version 5.2.0), and “dmetar” (version 0.0.9000) (Viechtbauer, 2010; Team, 2021; Sara et al., 2019). Given the expected heterogeneity across studies due to likely variations in methodologies and populations, a random-effects model was employed to compute the overall prevalence of *Salmonella* pathogen and antimicrobial-resistant *Salmonella* isolates in wild bird samples. The prevalence of *Salmonella* was estimated by dividing the number of *Salmonella*-positive samples by the total sample size. The prevalence of AMR *Salmonella* isolates was calculated by dividing the number of resistant isolates by the total number of isolates subjected to antibiotic susceptibility testing. The data were initially transformed to satisfy the assumption of normality using the Freeman-Tukey Double Arcsine transformation method (Freeman and Tukey, 1950). The overall prevalence rates and associated 95% confidence intervals (CI) were subsequently computed and converted back into percentages for clarity of interpretation. Subsequently, the DerSimonian and Laird (DL) method (DerSimonian and Laird, 1986) was applied to pool the data and estimate the between-study variance (*f*^2^). The DL method operates under the assumption that true effect sizes vary across studies, making it particularly suitable for analyzing heterogeneous datasets (Ren et al., 2024). Furthermore, this method provides a weighted average of the effect sizes, with studies with larger sample sizes contributing more to the overall estimate (Sidik and Jonkman, 2002). Subsequently, the between-study heterogeneity was evaluated using Cochran's Q-statistic (χ^2^) and *I*^2^ statistic, the proportion of total variance across studies due to true heterogeneity rather than sampling error (Higgins et al., 2003). Between-study heterogeneity was considered significantly high if the *I*^2^ was higher than 50% and the Q test yielded a *p*-value less than 0.05. To evaluate the impact of each individual study on the pooled prevalence and identify studies that may disproportionately affect the final effect size estimate, a sensitivity analysis was implemented using the leave-one-out approach (Wang, 2017).

To examine the underlying sources of any between-study heterogeneity, comprehensive moderator analyses encompassing both subgroup and meta-regression analyses were conducted. For the prevalence of antimicrobial-resistant *Salmonella* serovars, a subgroup analysis was conducted using the country, continent, sample source, antibiotic type, and susceptibility testing method. Conversely, the subgroup analysis of the overall prevalence of *Salmonella* pathogens in wild bird samples involved variables such as country, continent, bird species, migration status of the bird (i.e., migratory, semi-migratory, or non-migratory), sample type, sample source, and detection method. Univariate meta-regression was conducted with the publication year as the continuous moderator variable to explore potential temporal trends in *Salmonella* prevalence and AMR status in wild bird populations. Publication bias was visually appraised based on the symmetry of the funnel plots, and further confirmed using Egger's regression test (Sabarimurugan et al., 2019). When publication bias was verified, the Duval and Tweedie trim-and-fill method (Duval and Tweedie, 2000) was employed to estimate the unbiased prevalence by imputing potentially missing studies in the funnel plot.

## 3 Results

### 3.1 Search results

**Figure 1:**
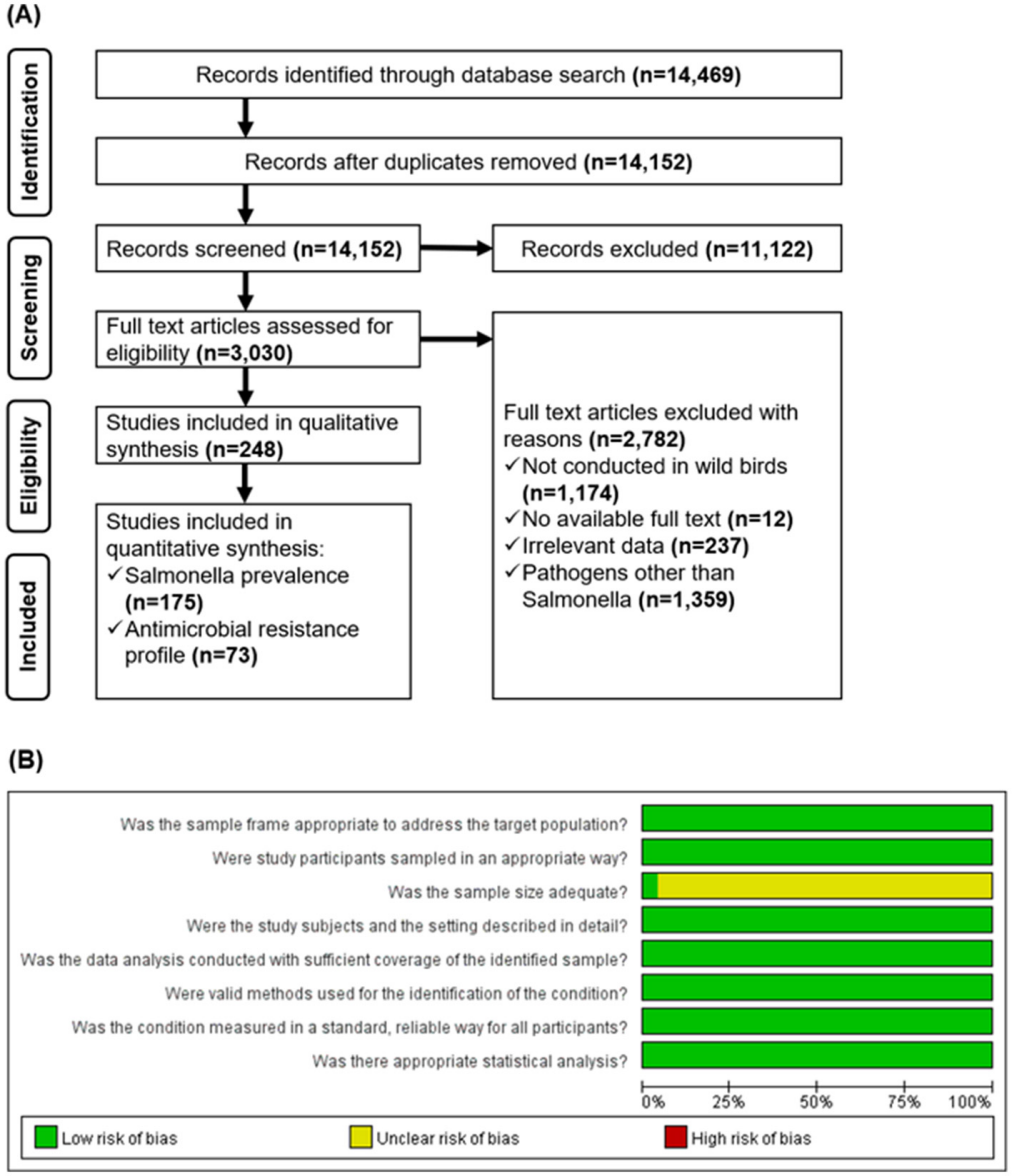
The preferred reporting items for systematic review and meta-analysis (PRISMA) flow diagram depicting study selection for the systematic review and meta-analysis of the prevalence of *Salmonella* spp. and their AMR patterns in global wild bird populations **(A)**. Risk of bias assessment of the eligible studies using the Joanna Briggs Institute (JBI) critical appraisal tools for prevalence studies **(B)**. Green and yellow denotes a low and unclear risk of bias, respectively.

In total, 14,469 full-text studies were identified through a systematic literature search of electronic databases and additional sources. After eliminating 317 duplicates, 14,152 studies were retained for title and abstract evaluation. Of these, 11,122 studies were excluded as they failed to meet the eligibility criteria, resulting in 3,030 studies for full-text screening. During this stage, 2,782 publications were systematically excluded for the following reasons: not conducted on wild birds (1,174 studies), no full text available (12 studies), data deemed irrelevant (237 studies), and studies focusing on pathogens other than *Salmonella* (1,359 studies). Ultimately, 248 studies satisfied the inclusion criteria and were therefore included in both the qualitative (systematic review) and quantitative (meta-analysis) analyses. Overall, 175 studies were included in the analysis of the general prevalence of *Salmonella* spp., whereas 73 were included in the assessment of AMR prevalence among *Salmonella* isolates. The numbers of studies retrieved at each stage of the screening process are shown in Figure 1A.

### 3.2 Assessment of the risk of bias

The risk of bias (RoB) assessment of 175 eligible studies demonstrated a low RoB across most of the evaluated domains (Figure 1B). Specifically, all studies showed a minimal risk of bias in critical items, including the appropriateness of the sampling framework to address the target population, sampling methodology of the wild birds from different sample sources, comprehensive description of the birds and their living environments, sufficiency of data analysis coverage, use of valid methods to identify *Salmonella* infection in birds, standardized and reliable measurement of *Salmonella* infection in all birds tested, and the implementation of appropriate statistical analyses to compute the prevalence of *Salmonella* spp. and other variables. The assessment further revealed some uncertainty regarding the adequacy of the sample size, where 168/175 studies (96%) had an unclear risk of bias, while only 7/175 studies (4%) had a low risk of bias. This suggests that the majority of studies did not provide sufficient information to conclusively determine sample size adequacy, which could potentially affect the robustness of their findings. The RoB assessment affirmed the credibility of the synthesized evidence on the general and AMR prevalence of *Salmonella* spp. in wild bird populations with minimal concerns about bias that would undermine the validity of the meta-analytic findings.

### 3.3 Characteristics of eligible studies

**Figure 2:**
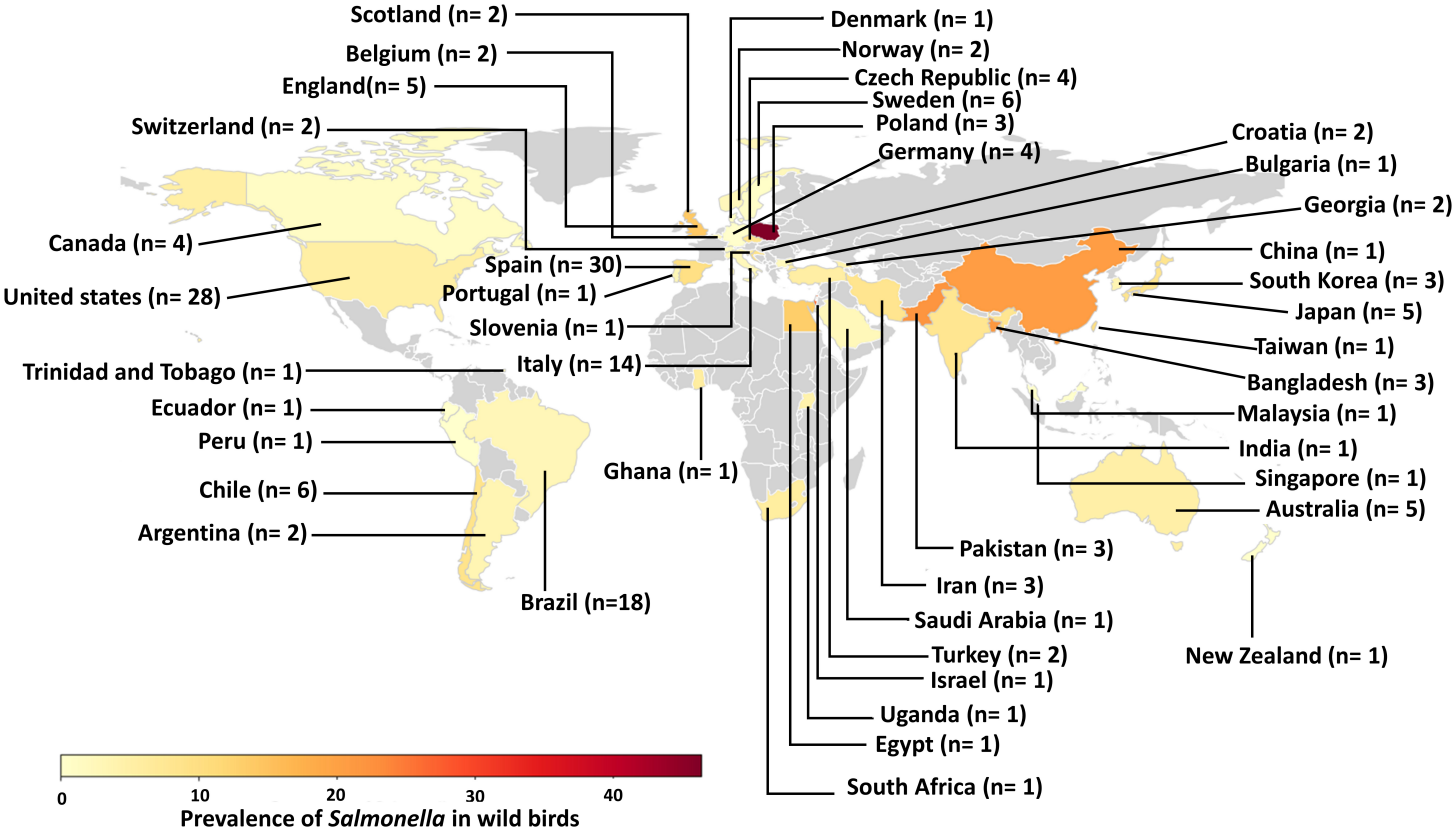
Global distribution of studies reporting on the prevalence and antimicrobial resistance profiles of *Salmonella* serovars in wild birds from 2000 to 2025. The intensity of color shading reflects the prevalence levels, ranging from low (light yellow) to high (dark red). The notation “*n*” indicates the number of studies conducted within each country.

Of the 248 studies included in the meta-analysis, 175 investigated the general prevalence of *Salmonella* (Wiederkehr et al., 2024; Aung et al., 2019; Card et al., 2023; Espunyes et al., 2022; Teixeira Campos et al., 2025; Manzanares-Pedrosa et al., 2025; Soltan Dallal et al., 2024; Cabodevilla et al., 2024; Chu et al., 2024; Corradini et al., 2024; Vogler et al., 2024; Mafizur et al., 2024; Martín-Vélez et al., 2024; Mousavinezhad et al., 2024a; Sánchez-Cano et al., 2024; Tallon et al., 2024; De Castro Teixeira et al., 2024; Batista et al., 2023; Belo et al., 2023; López et al., 2023; Musa et al., 2023; Saeed et al., 2023a,b; Smith et al., 2023; Nagamori et al., 2022; Russo et al., 2022; Smith et al., 2022b; Smith H. G. et al., 2020; Sahan Yapicier et al., 2022; Antilles et al., 2021; Blanco and Díaz de Tuesta, 2021; Cardoso et al., 2021; Choi et al., 2021; Cummings et al., 2021; Ebani et al., 2021; Elsohaby et al., 2021; Malekian et al., 2021; Modupe et al., 2021; Russo et al., 2021; Saiful Islam et al., 2021; Vogler et al., 2021; de Souza et al., 2020; Dos Santos et al., 2020; Fonseca et al., 2020; Martín-Maldonado et al., 2020; Miller et al., 2020; Navarro-Gonzalez et al., 2020; Perez-Sancho et al., 2020; Plaza-Rodríguez et al., 2020; Sharif et al., 2020; Suárez-Pérez et al., 2020; Tardone et al., 2020; Valdebenito et al., 2020; Wei et al., 2020; Navarro et al., 2019; Plaza et al., 2019; Fuentes-Castillo et al., 2019; Martín-Maldonado et al., 2019; Söderlund et al., 2019; Thierfelder et al., 2019; Vogt et al., 2019a,b; De Lucia et al., 2018; de Oliveira et al., 2018; Gargiulo et al., 2018; Horn et al., 2018; MacDonald et al., 2018; Machado et al., 2018; Milton et al., 2018; Najdenski et al., 2018; Okamura et al., 2018; Rodríguez et al., 2018; Brobey et al., 2017; Grigar et al., 2017; Migura-Garcia et al., 2017; Schmitz et al., 2017; Afema and Sischo, 2016; Camacho et al., 2016; Dolejska et al., 2016; Grigar et al., 2016; Haesendonck et al., 2016; Hernandez et al., 2016; Huang et al., 2016; Jurado-Tarifa et al., 2016; Konicek et al., 2016; Marenzoni et al., 2016; Masarikova et al., 2016; Matias et al., 2016; Rouffaer et al., 2016; Rubini et al., 2016; Antilles et al., 2015a,b; Braconaro et al., 2015; Dougnac et al., 2015; Janecko et al., 2015; Krawiec et al., 2015; Lopes et al., 2015; Molina-López et al., 2015; Callaway et al., 2014; Dipineto et al., 2014; Gargiulo et al., 2014; Jay-Russell et al., 2014; Krawiec et al., 2014; Marin et al., 2014; Pao et al., 2014; Sulzner et al., 2014; Andrés et al., 2013; Fresno et al., 2013; Giovannini et al., 2013; Marques et al., 2013; Osman et al., 2013; Stenkat et al., 2013; Tel et al., 2013; Cho et al., 2012; Hamer et al., 2012; Hoque et al., 2012; Mtshali, 2012; Rodríguez et al., 2012; Velarde et al., 2012; Foti et al., 2011; Lawson et al., 2011; Molina-Lopez et al., 2011; Ruiz-de-Castañeda et al., 2011; Butron and Brightsmith, 2010; Kitadai et al., 2010; Lawson et al., 2010; Marietto-Gonçalves et al., 2010a,b; Mirzaie et al., 2010; Ramos et al., 2010; Sousa et al., 2010; Dolejská et al., 2009; Foti et al., 2009; Iveson et al., 2009; Ortiz–Catedral et al., 2009; Allgayer et al., 2008; Bogomolni et al., 2008; Gionechetti et al., 2008; Haemig et al., 2008; Hughes et al., 2008; Jang et al., 2008; Kinzelman et al., 2008; Skov et al., 2008; Chen et al., 2008; Ganapathy et al., 2007; Jijón et al., 2007; Kobayashi et al., 2007; Literák et al., 2007; Palmgren et al., 2006; Pedersen et al., 2006; Dobbin et al., 2005; Lillehaug et al., 2005; Pennycott et al., 2005; Steele et al., 2005; Tanaka et al., 2005; Dovc et al., 2004; Fallacara et al., 2004; Padilla et al., 2004; Palmgren et al., 2004; Vlahović et al., 2004; Höfle et al., 2003; Reche et al., 2003a,b; Refsum et al., 2003; Vučemilo et al., 2003; Wahlström et al., 2003; Kirk et al., 2002; Pennycott et al., 2002; Smith et al., 2002; Fallacara et al., 2001; Maeda et al., 2001; Craven et al., 2000; Gopee et al., 2000; Palmgren et al., 2000; Smith O. M. et al., 2020; Smith et al., 2022a) and 73 assessed the AMR status of *Salmonella* serovars in wild birds (Wiederkehr et al., 2024; Aung et al., 2019; Card et al., 2023; Espunyes et al., 2022; Teixeira Campos et al., 2025; Soltan Dallal et al., 2024; Corradini et al., 2024; Vogler et al., 2024; Batista et al., 2023; Saeed et al., 2023a,b; Russo et al., 2022; Smith et al., 2022b; Smith H. G. et al., 2020; Sahan Yapicier et al., 2022; Cardoso et al., 2021; Elsohaby et al., 2021; Russo et al., 2021; Saiful Islam et al., 2021; Vogler et al., 2021; Dos Santos et al., 2020; Martín-Maldonado et al., 2020; Sharif et al., 2020; Smith et al., 2020c; Suárez-Pérez et al., 2020; Tardone et al., 2020; Wei et al., 2020; Fuentes-Castillo et al., 2019; Martín-Maldonado et al., 2019; Vogt et al., 2019a; de Oliveira et al., 2018; Najdenski et al., 2018; Rodríguez et al., 2018; Grigar et al., 2017; Migura-Garcia et al., 2017; Camacho et al., 2016; Dolejska et al., 2016; Grigar et al., 2016; Jurado-Tarifa et al., 2016; Masarikova et al., 2016; Matias et al., 2016; Rubini et al., 2016; Dougnac et al., 2015; Janecko et al., 2015; Lopes et al., 2015; Molina-López et al., 2015; Sulzner et al., 2014; Andrés et al., 2013; Fresno et al., 2013; Osman et al., 2013; Molina-Lopez et al., 2011; Kitadai et al., 2010; Marietto-Gonçalves et al., 2010a,b; Mirzaie et al., 2010; Dolejská et al., 2009; Gionechetti et al., 2008; Hughes et al., 2008; Chen et al., 2008; Jijón et al., 2007; Kobayashi et al., 2007; Literák et al., 2007; Palmgren et al., 2006; Dobbin et al., 2005; Steele et al., 2005; Fallacara et al., 2004; Smith et al., 2002; Fallacara et al., 2001; Gopee et al., 2000; Palmgren et al., 2000; Mousavinezhad et al., 2024b; La Tela et al., 2021; Krawiec et al., 2017; Mather et al., 2016; Reche et al., 2003a,b). The selected studies were conducted in 44 countries, including six from South America, 17 from Europe, two from North America, 13 from Asia, two from Oceania, and four from Africa, between 2000 and 2025. Figure 2 illustrates the global distribution of published studies on the prevalence and AMR patterns of *Salmonella* isolates in wild birds between 2000 and 2025. Studies assessing the prevalence of *Salmonella* in wild birds used 73,087 samples. Among these, 66,118 were sourced from free-living wild birds, 2,958 from rescue and rehabilitation centers, 1,139 from zoos, 607 from captive birds, 543 from veterinary hospitals, 121 from research centers, 82 from conservation centers, and 60 from diagnostic centers. The AMR prevalence analysis examined 1,339 *Salmonella* isolates, including 1,112 from free-living wild birds, 105 from rescue and rehabilitation centers, 50 from zoos, 18 from pigeon lofts, 10 from research centers, and 2 from diagnostic centers; the sources of 42 *Salmonella* isolates were not specified.

Regarding antimicrobial susceptibility testing (AST), the disk diffusion method was the most commonly used approach, employed in 52 studies (72%), followed by the broth microdilution method in 17 studies (24%), the VITEK 2 Compact system in 2 studies (3%), and the agar dilution method in 1 study (1%). To interpret resistance levels, several breakpoint standards were used, including the Clinical and Laboratory Standards Institute (CLSI) guidelines (43 studies), the European Committee on Antimicrobial Susceptibility Testing (EUCAST) guidelines (10 studies), the National Committee on Clinical Laboratory Standards (NCCLS) guidelines (7 studies), the Swedish Reference Group for Antibiotic and Resistance Methods (RAF-M) guidelines (2 studies), the Canadian Integrated Program for Antimicrobial Resistance Surveillance (CIPARS) guidelines (1 study), the British Society for Antimicrobial Chemotherapy (BSAC) guidelines (1 study), the Danish Integrated Antimicrobial Resistance Monitoring and Research Program (DANMAP 2001) guidelines (1 study), and the Japanese Society of Chemotherapy (JSC) guidelines (1 study). Six studies did not report the interpretive standard used. Overall, a total of 85 antimicrobial agents belonging to 21 antimicrobial classes were tested: aminoglycosides, carbapenems, cephalosporins, diaminopyrimidines, fluoroquinolones, folate pathway inhibitors, glycopeptides, glycylcyclines, lincosamides, lincosamide–aminocyclitols, macrolides, monobactams, penicillins, penicillins combined with β-lactamase inhibitors, phenols, phosphonics, polymyxins, polypeptides, quinolones, sulfonamides, and tetracyclines. Collectively, these data provide a comprehensive global overview delineating the geographical distribution, temporal patterns, and methodological heterogeneity characterizing the general AMR prevalence of *Salmonella* spp. in wild bird populations. The detailed characteristics of the studies included in the meta-analysis are presented in Supplementary files 2, 3.

### 3.4 Meta-analysis

#### 3.4.1 General prevalence estimate

**Figure 3:**
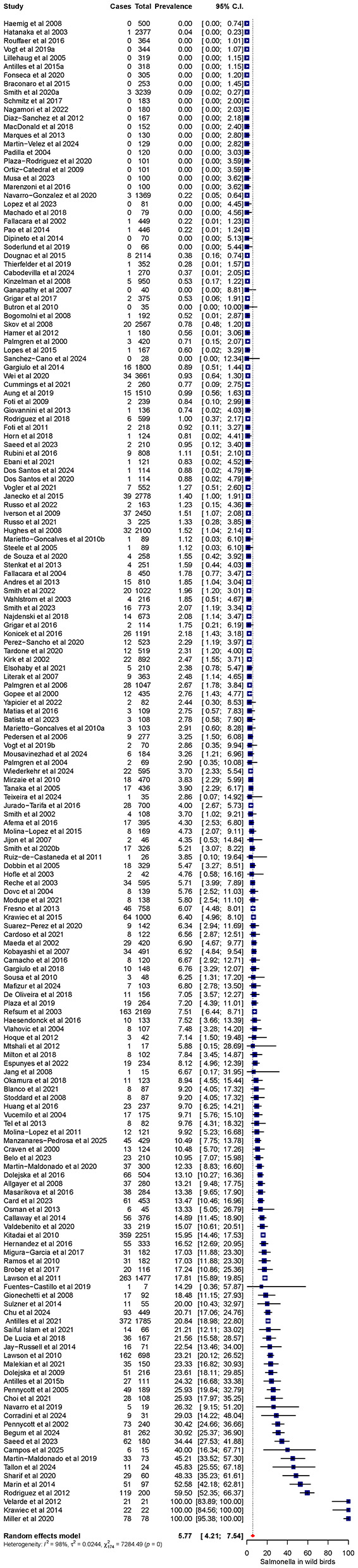
Forest plot of 175 studies assessing the general prevalence of *Salmonella* spp. in wild birds. The blue squares represent the prevalence estimates for each individual study with their 95% confidence intervals (CIs), while the red diamond indicates the pooled prevalence estimate.

The pooled prevalence of *Salmonella* spp. in wild bird populations was 5.77%, with a 95% CI of 4.21 to 7.54 (Figure 3). Significant between-study heterogeneity was observed (*I*^2^ = 98%, *p* < 0.001), indicating substantial variability among individual studies. A leave-one-out sensitivity analysis identified six studies (Miller et al., 2020; Sharif et al., 2020; Krawiec et al., 2014; Marin et al., 2014; Rodríguez et al., 2012; Velarde et al., 2012) as potential outliers due to their extreme prevalence estimates (Supplementary file 4). To investigate their influence on the pooled prevalence, the analysis was repeated after excluding these studies. The pooled prevalence shifted from 5.77% (95% CI: 4.21–7.54%) to 4.47% (95% CI: 3.56–5.47%). However, between-study heterogeneity remained largely unchanged (*I*^2^ = 98% vs. 97%), suggesting that the overall variability across studies persisted despite the omission of outliers. To explore potential sources of heterogeneity, subgroup analyses were conducted based on eight predefined covariates: country of origin, bird species, continent, sample source, detection method, migration behavior, isolated serovars, and sample type. The results indicated that country of origin, bird species, serovar, and sample type were significantly associated with between-study heterogeneity (*p* < 0.01). At the country level, the highest prevalence of *Salmonella* was reported in Poland (46.4%; 95% CI: 23.75–69.83%), followed by Scotland (28.16%; 95% CI: 11.01–49.45%) and Israel (25.93%; 95% CI: 18.06–34.65%; Table 1). In contrast, no *Salmonella* isolates were reported in studies conducted in Peru, New Zealand, Malaysia, or Ecuador. When stratified by bird species, Accipitriformes exhibited the highest pooled prevalence (6.85%; 95% CI: 2.78–12.01%), followed by Charadriiformes (6.15%; 95% CI: 3.59–9.17%) and Pelecaniformes (5.98%; 95% CI: 0.93–13.55%), whereas Apodiformes, Gruiformes, and Anseriformes showed no detected infections (i.e., 0% prevalence; Table 2).

**Table 1:** Subgroup analysis of the prevalence of *Salmonella* spp. in wild bird populations based on the country.

S/N	Country	Prevalence (%)	95% confidence interval (CI)	S/N	Country	Prevalence (%)	95% confidence interval (CI)
1.	Poland	46.4	23.75–69.83	23.	Uganda	4.3	2.50–6.55
2.	Scotland	28.16	11.01–49.45	24.	Georgia	4.09	0.00–17.16
3.	Israel	25.93	18.06–34.65	25.	Argentina	3.39	0.00–15.49
4.	Pakistan	22.05	8.90–38.90	26.	South Korea	3.29	0.00–14.45
5.	Bangladesh	21.38	8.57–37.90	27.	Brazil	3.01	0.77–6.36
6.	China	20.71	17.08–24.59	28.	Portugal	2.78	0.35–6.92
7.	England	14.06	5.21–26.26	29.	Trinidad	2.76	1.40–4.53
8.	Egypt	13.33	4.71–25.06	30.	Saudi Arabia	2.38	0.67–4.97
9.	Taiwan	9.70	6.23–13.83	31.	Norway	2.29	0.00–13.07
10.	Spain	9.63	6.32–13.50	32.	Italy	2.27	0.23–5.83
11.	Chile	8.63	2.27–17.90	33.	Bulgaria	2.08	1.12–3.31
12.	Croatia	8.59	0.33–24.96	34.	Belgium	2.05	0.00–13.00
13.	Czech Republic	8.47	1.91–18.92	35.	Canada	1.19	0.00–7.31
14.	Iran	8.32	1.14–20.80	36.	Switzerland	1.00	0.00–10.28
15.	Japan	8.15	2.22–17.23	37.	Singapore	0.99	0.55–1.56
16.	India	7.84	3.30–13.97	38.	Denmark	0.78	0.47–1.16
17.	South Africa	5.88	0.00–23.54	39.	Sweden	0.71	0.00–4.81
18.	Ghana	5.80	2.42–10.41	40.	Germany	0.27	0.00–4.83
19.	Slovenia	5.76	2.40–10.33	41.	Peru	0.00	0.00–4.86
20.	Turkey	5.58	0.00–20.73	42.	New Zealand	0.00	0.00–1.70
21.	USA	5.18	2.78–8.20	43.	Malaysia	0.00	0.00–4.25
22.	Australia	4.89	0.54–12.75	44.	Ecuador	0.00	0.00–1.43

**Table 2:** Subgroup analysis of *Salmonella* prevalence in wild birds based on seven potential covariates.

Variable	Category	No. studies	Prevalence estimate (95%CI)	Heterogeneity		Test for subgroup differences
				*I* ^2^	*p*−*value*^d^	*p-*value^e^
Continent	South America	29	3.57% (1.49–6.34)	96	<0.01	= 0.09
	Europe	81	6.14% (4.48–8.02)	98	<0.01	
	North America	32	4.55% (2.41–7.27)	97	<0.01	
	Asia	24	10.13% (6.46–14.48)	98	<0.01	
	Oceania	6	3.66% (0.23–10.22)	96	<0.01	
	Africa	4	6.66% (0.38–17.98)	43	= 0.15	
Sample source	Wild	138	5.99% (4.72–7.38)	98	<0.01	= 0.35
	Rescue and Rehabilitation center	29	5.39% (2.82–8.63)	96	<0.01	
	Captivity	10	7.70% (2.77–14.50)	90	<0.01	
	Diagnostic center	4	0.82% (0.00–7.20)	0	= 0.77	
Detection method	Biochemical tests	129	5.04% (3.81–6.42)	97	<0.01	= 0.09
	Molecular tests	28	9.64% (6.18–13.72)	99	<0.01	
	Metabarcoding	1	0.37% (0.00–1.58)	-	-	
	MALDI-TOF-MS^a^	6	5.14% (0.35–13.48)	96	<0.01	
	Serotyping	11	7.58% (3.21–13.51)	99	<0.01	
Migration behavior	Migratory	80	3.45% (1.95–5.24)	97	<0.01	= 0.45
	Semi-migratory	39	4.13% (1.61–7.39)	95	<0.01	
	Non-migratory	91	4.88% (3.29–6.70)	97	<0.01	
	Mixed samples	41	4.26% (2.36–6.60)	96	<0.01	
Sample type	Cloacal swabs	99	4.79% (3.39–6.39)	97	<0.01	< 0.01
	Feces	62	6.11% (4.23–8.30)	98	<0.01	
	Blood	1	46.81% (32.64–61.23)	-	-	
	Cecal content	2	11.85% (1.39–29.99)	65	=0.09	
	Internal organs	15	10.40% (5.72–16.20)	99	<0.01	
	Oral swabs	3	4.44% (0.00–18.64)	94	<0.01	
	Intestinal content	2	50.46% (13.22–87.45)	0	=0.60	
	Mixed samples	7	5.32% (0.62–13.02%)	96	<0.01	
Bird species	Pelecaniformes	22	5.98% (0.93–13.55%)	27	=0.12	< 0.01
	Charadriiformes	42	6.15% (3.59–9.17%)	97	<0.01	
	Passeriformes	47	4.22% (2.43–6.38%)	97	<0.01	
	Anseriformes	37	0.00% (0.00–0.87%)	92	<0.01	
	Ciconiiformes	11	3.77% (0.07–10.63%)	66	<0.01	
	Columbiformes	36	0.47% (0.00–2.45%)	80	<0.01	
	Psittaciformes	17	0.02% (0.00–2.30%)	85	<0.01	
	Accipitriformes	25	6.85% (2.78–12.01%)	88	<0.01	
	Galliformes	16	0.03% (0.00–3.14%)	90	<0.01	
	Falconiformes	16	0.36% (0.00–4.43%)	0	= 0.67	
	Apodiformes	9	0.00% (0.00–1.28%)	0	= 0.99	
	Piciformes	11	0.06% (0.00–7.20%)	25	= 20	
	Gruiformes	15	0.00% (0.00–3.99%)	72	<0.01	
	Strigiformes	21	5.29% (1.26–10.95%)	96	<0.01	
	Other species	76	0.00% (0.00–0.72%)	94	<0.01	
Serovars	S. Typhimurium	65	4.12% (2.99–5.41%)	98	<0.01	< 0.01
	S. Enteritidis	30	1.42% (0.51–2.71%)	93	<0.01	
	S. Virchow	9	0.41% (0.00–2.12%)	74	<0.01	
	S. Infantis	14	0.26% (0.00–1.67%)	78	<0.01	
	Other serovars^b^	93	4.13% (3.14–5.23%)	97	<0.01	
	*Salmonella* spp.^c^	43	6.51% (4.72–8.53%)	98	<0.01	

^a^ Matrix-assisted laser desorption/ionization time-of-flight mass spectrometry.

^b^ Data from the remaining serovars were combined for the analysis.

^c^ Prevalence estimate was reported at the genus level because serovar-level data were not available.

^d^ A statistically significant p-value (p < 0.05) indicates the presence of significant between-study heterogeneity.

^e^ A statistically significant p-value (p < 0.05) indicates a significant difference in prevalence estimates between subgroups.

Regarding the sample type, the highest prevalence estimates were observed in intestinal contents (50.46%; 95% CI: 13.22–87.45%) and blood samples (46.81%; 95% CI: 32.64–61.23%), although these estimates were based on only one or two studies. Among commonly used samples types, cloacal swabs (99 studies) demonstrated a pooled prevalence of 4.79% (95% CI: 3.39–6.39%), fecal samples (62 studies) 6.11% (95% CI: 4.23–8.30%), and internal organs (15 studies) showed a prevalence of 10.40 % (95% CI: 5.72–16.20 %; Table 2). Serovar-specific analysis revealed that *S*. Typhimurium had the highest prevalence (4.12%; 95% CI: 2.99–5.41%), followed by *S*. Enteritidis (1.42%; 95% CI: 0.51–2.71%). Other serovars were detected at substantially lower levels, including *S*. Virchow (0.41%; 95% CI: 0.00–2.12%) and *S*. Infantis (0.26%; 95% CI: 0.00–1.67%). Additionally, studies that reported prevalence only at the genus level demonstrated a pooled prevalence of 6.51% (95% CI: 4.72–8.53%), while other serovars collectively accounted for 4.13% (95% CI: 3.14–5.23%). Other examined covariates, including continent, sample source, detection method, and bird migration behavior, showed variation among subgroups but were not statistically significant (*P* > 0.05; Table 2). These findings further emphasize the marked geographical, taxonomic, and methodological influences on the pooled prevalence estimates, thereby underscoring the need for standardized surveillance methods to accurately assess *Salmonella* infection in wild bird populations.

#### 3.4.2 AMR prevalence estimates

Analysis of the AMR profile of *Salmonella* isolates from wild birds incorporated data from 73 eligible studies examining resistance rates against 85 antimicrobials classified into 21 medically important antimicrobial groups. According to the World Health Organization (WHO), medically important antimicrobials can be categorized into three groups based on different prioritization factors: critically important, highly important, and important (World Health Organization, 2017). Regarding critically important antimicrobials (CIAs), *Salmonella* spp. isolated from wild birds showed the highest resistance to macrolides (29.64%; 95% CI: 6.98–57.43%), followed by monobactams (22.89%; 95% CI: 0.00–100%) and penicillins (14.71%; 95% CI: 8.07–22.41%). In contrast, the lowest resistance levels (i.e., highest susceptibility) were observed for penicillins combined with β-lactamase inhibitors (0%; 95% CI: 0.00–0.14%), phosphonics (0%; 95% CI: 0.00–0.13%), fluoroquinolones (0.37%; 95% CI: 0.00–1.94%), and carbapenems (0.39%; 95% CI: 0.00–4.95%). Among highly important antimicrobials, the highest resistance was observed to lincosamide-aminocyclitol combinations (28.69%; 95% CI: 0.00–100%), followed by tetracyclines (15.90%; 95% CI: 8.32–24.77%) and sulfonamides (14.51%; 95% CI: 0.00–43.60) whereas the lowest resistance was detected for folate pathway inhibitors (0.77%; 95% CI: 0.00–3.57%) and phenols (1.05%; 95% CI: 0.00–3.55%). Among important antimicrobials, polypeptides were the only antimicrobial class tested in this category and exhibited a substantially high resistance rate of 100% (95% CI: 98.00–100%). Furthermore, resistance to glycopeptides (91.98%; 95% CI: 56.31–100) and lincosamides (93.70%; 95% CI: 52.20–100) was also markedly high; however, these findings reflect the intrinsic resistance of *Salmonella spp*. and should not be interpreted as evidence of emerging or acquired antimicrobial resistance in wild bird populations. Table 3 presents a comprehensive summary of the results derived from the meta-analysis and meta-regression analyses of AMR prevalence in *Salmonella* isolates categorized by the antimicrobial group. Significant heterogeneity (*I*^2^ > 50%, *p* < 0.01) was a common finding across most antimicrobial classes, indicating substantial variation in resistance rates across studies. A univariate meta-regression analysis was conducted to assess temporal trends in the prevalence of AMR *Salmonella* serovars in wild bird populations, with the publication year serving as a covariate (Table 3). The analysis demonstrated a statistically significant increase in AMR over time for several antibiotic classes, including penicillins with β-lactamase inhibitors (*R*^2^ = 66.75%, *p* < 0.01), phenicols (*R*^2^ = 20.94%, *p* = 0.03), fluoroquinolones (*R*^2^ = 19.21%, *p* = 0.003), aminoglycosides (*R*^2^ = 11.05%, *p* =0.01), and macrolides (*R*^2^ = 4.60%, *p* = 0.02).

**Table 3:** Meta-analysis and meta-regression of the prevalence of AMR of *Salmonella* isolates from wild birds for each antimicrobial group.

Medical importance	Antibiotic class	No. studies	Prevalence (95% CI)	Heterogeneity		Meta-regression (based on the publication year)	
				*I* ^2^	*p*-value^b^	*R* ^2^	*p*-value^c^
Critically important	Aminoglycosides	71	5.09% (2.07–8.96)	85	<0.01	11.05%	= 0.01
	Carbapenems	21	0.39% (0.00–4.95)	79	<0.01	0.00%	= 0.89
	Cephalosporins	61	1.38% (0.04–4.06)	76	<0.01	0.78%	= 0.46
	Fluoroquinolones	65	0.37% (0.00–1.94)	67	<0.01	19.21%	= 0.003
	Glycopeptides^a^	5	91.98% (56.31–100)	63	= 0.03	-^d^	-
	Glycylcyclines	6	0.83% (0.00–7.04)	28	= 0.23	-	-
	Macrolides	21	29.64% (6.98–57.43)	94	<0.01	4.60%	= 0.02
	Monobactams	2	22.89% (0.00–100)	94	<0.01	-	-
	Penicillins	67	14.71% (8.07–22.41)	87	<0.01	11.34%	= 0.44
	Penicillins/b-lactamase inhibitors	34	0.00% (0.00–0.14)	36	<0.01	66.75%	<0.01
	Phosphonics	4	0.00% (0.00–0.13)	0	= 0.83	-	-
	Polymyxins	20	4.45% (0.27–11.46)	80	<0.01	3.72%	= 0.72
	Quinolones	43	8.38% (2.20–16.82)	86	<0.01	2.16%	= 0.75
Highly important	Diaminopyrimidines	19	3.39% (0.00–12.10)	63	<0.01	0.00%	= 0.93
	Folate pathway inhibitors	47	0.77% (0.00–3.57)	68	<0.01	25.74%	= 0.07
	Lincosamides^a^	7	93.70% (52.20–100)	93	<0.01	-	-
	Lincosamides-Aminocyclitols	2	28.69% (0.00–100)	35	= 0.21	-	-
	Phenicols	57	1.05% (0.00–3.55)	61	<0.01	20.94%	= 0.03
	Sulfonamides	14	14.51% (0.00–43.60)	93	<0.01	0.00%	= 0.36
	Tetracyclines	62	15.90% (8.32–24.77)	92	<0.01	13.44%	= 0.36
Important	Polypeptides	3	100% (98.00–100)	0	= 0.83	-	-

^a^ High resistance reflects intrinsic resistance of Salmonella to these antimicrobials, not acquired resistance.

^b^ A statistically significant p-value (p < 0.05) indicates the presence of significant between-study heterogeneity.

^c^ A statistically significant p-value (p < 0.05) indicates a significant association between the year of publication and the estimated prevalence.

^d^ Univariate meta-regression analysis was not conducted because the number of included studies was insufficient (< 10 studies).

**Figure 4:**
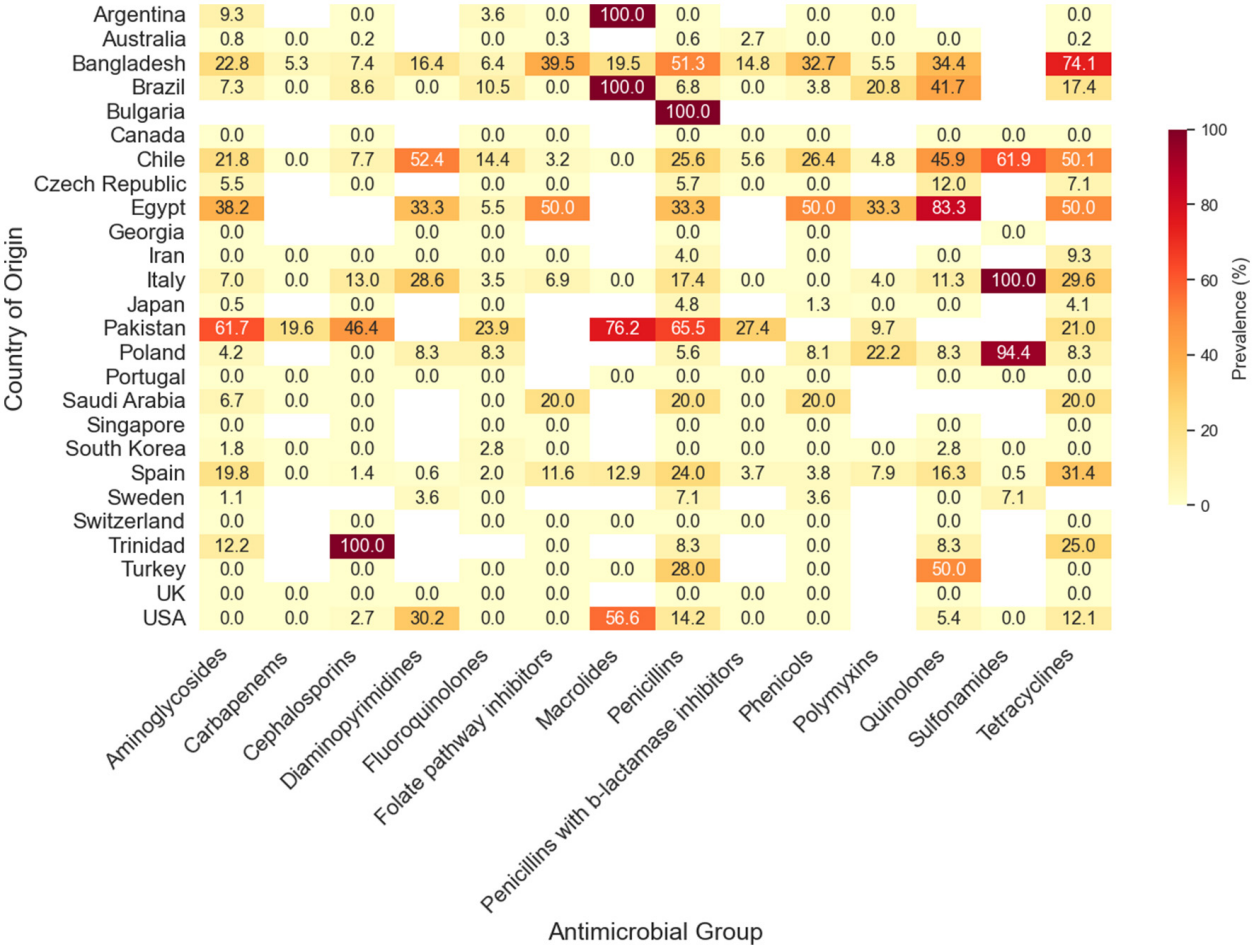
The prevalence of antimicrobial-resistant *Salmonella* isolates from global wild birds, disaggregated by antimicrobial group and country of origin. The y- and x-axes display the countries and antimicrobials, respectively. Values represent the AMR prevalence, with colors ranging from light yellow (0% prevalence) to dark red (100% prevalence). White boxes indicate countries where no studies were conducted for specific antimicrobial groups.

**Figure 5:**
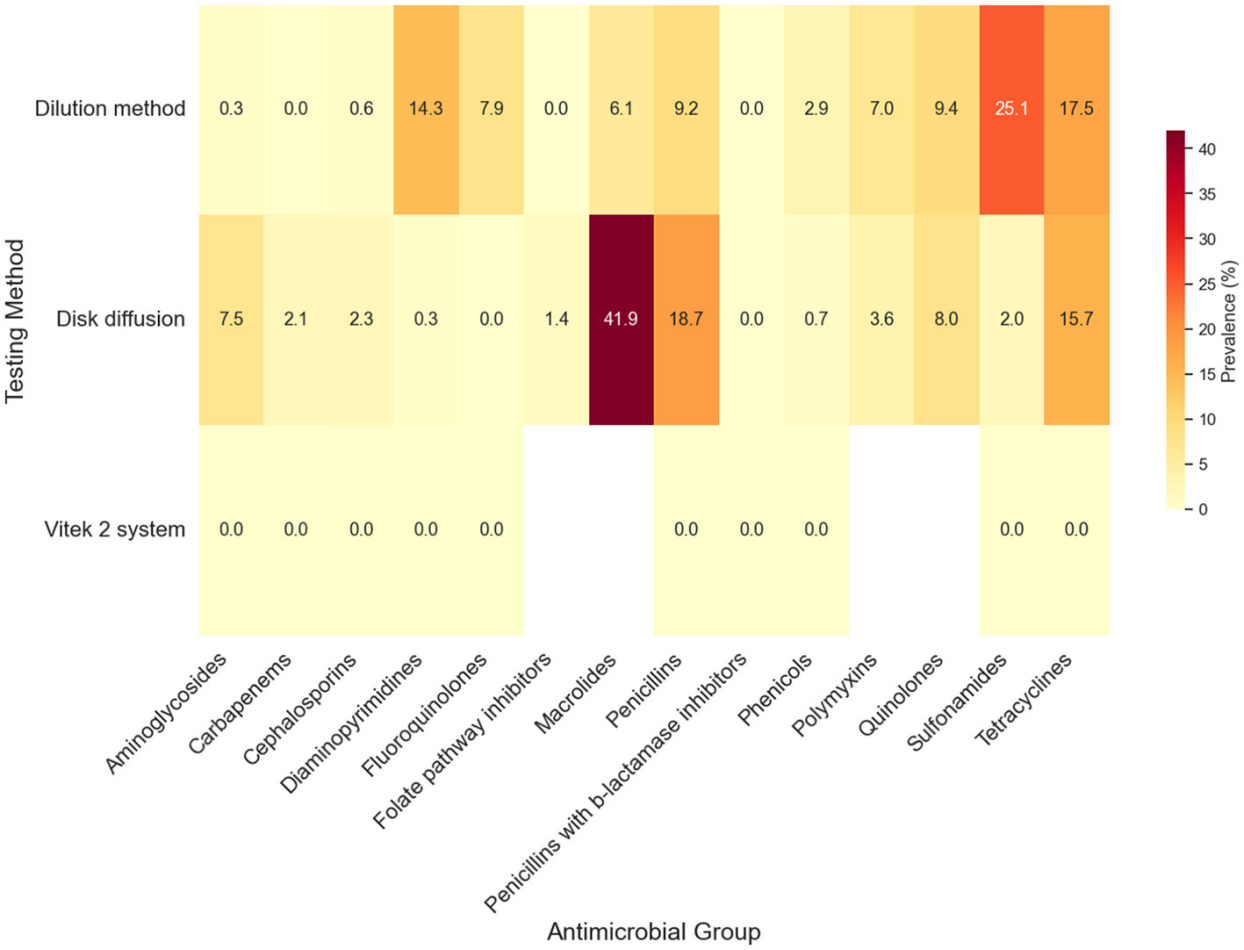
Subgroup analysis of the prevalence of antimicrobial-resistant *Salmonella* isolates in wild birds based on the susceptibility testing method. The testing methods are listed on the y-axis, while antimicrobial groups are displayed on the x-axis. Values represent the AMR prevalence, with colors ranging from light yellow (0% prevalence) to dark red (100% prevalence).

Subgroup analyses were conducted to investigate the potential sources of heterogeneity in AMR patterns among *Salmonella* serovars. Four putative moderators were assessed for their influence on observed resistance: country of origin, continent, AST method, and sample source. Based on the country, significant variation in AMR was evident across most antimicrobial groups (*p* < 0.01), particularly for aminoglycosides, cephalosporins, diaminopyrimidines, folate pathway inhibitors, penicillins, penicillin-β-lactamase inhibitor combinations, phenicols, and sulfonamides (Figure 4). Notably, one study conducted in Egypt reported exceptionally high resistance rates, with prevalence estimates reaching 83.3% for quinolones and 50% for phenicols and folate pathway inhibitors. These findings, however, should be interpreted cautiously, as they are based on limited evidence and may reflect local sampling conditions rather than the national resistance profile. Conversely, several countries, including Switzerland, Singapore, and Portugal, reported minimal to no detectable resistance across a broad range of antimicrobials (Figure 4). Resistance to CIAs, such as carbapenems, fluoroquinolones, and penicillins with β-lactamase inhibitors, remained rare or absent in most countries. The subgroup analysis indicated that AST method did not significantly influence the pooled AMR prevalence estimates across antimicrobial classes, as no statistically significant differences were observed between dilution methods, disk diffusion, and VITEK 2 compact system (Figure 5). These findings suggest that methodological variation in susceptibility testing was not a major contributor to heterogeneity in AMR prevalence among *Salmonella* isolates in wild bird populations.

**Figure 6:**
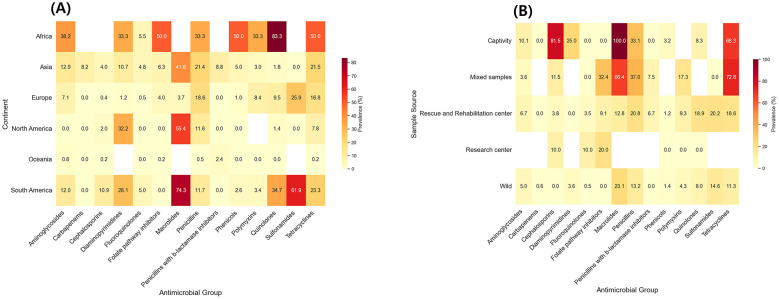
Subgroup analysis of the prevalence of antimicrobial-resistant *Salmonella* isolates in wild birds based on the continent **(A)** and sample source **(B)**. Values represent the AMR prevalence, with colors ranging from light yellow (0% prevalence) to dark red (100% prevalence). White spaces indicate that no studies were conducted for specific antimicrobial groups.

Furthermore, a statistically significant difference (*p* < 0.05) in AMR prevalence was detected across continents for several antimicrobial groups, including aminoglycosides, cephalosporins, diaminopyrimidines, and quinolones (Figure 6A). Most importantly, the highest prevalence rate for resistance to all these antibiotics, except for cephalosporins, was detected in African wild birds. These findings together underscore the highly heterogeneous global distribution of AMR in *Salmonella* isolates from wild birds. For carbapenems, cephalosporins, folate pathway inhibitors, and tetracyclines, the reported AMR rates significantly differed (*p* < 0.05) depending on the sample source, with samples collected from birds living in rescue and rehabilitation centers and captivity consistently showing the highest resistance rates (Figure 6B). These findings together highlight the complex and context-sensitive nature of AMR dissemination in global wild bird populations, thus emphasizing the imperative necessity to consider geological, methodological, and ecological variables in future surveillance initiatives. It is also important to note that subgroup and meta-regression analyses for antibiotic classes including glycopeptides, glycylcyclines, lincosamides, lincosamides-aminocyclitols, monobactams, phosphonics, polymyxins, and polypeptides, were not conducted due to an insufficient number of studies for robust statistical modeling. More detailed results of the subgroup analyses of the prevalence of AMR *Salmonella* serovars, along with their corresponding 95% confidence intervals, stratified by continent, AST method, and sample source, are provided in Supplementary file 5.

#### 3.4.3 Publication bias

**Figure 7:**
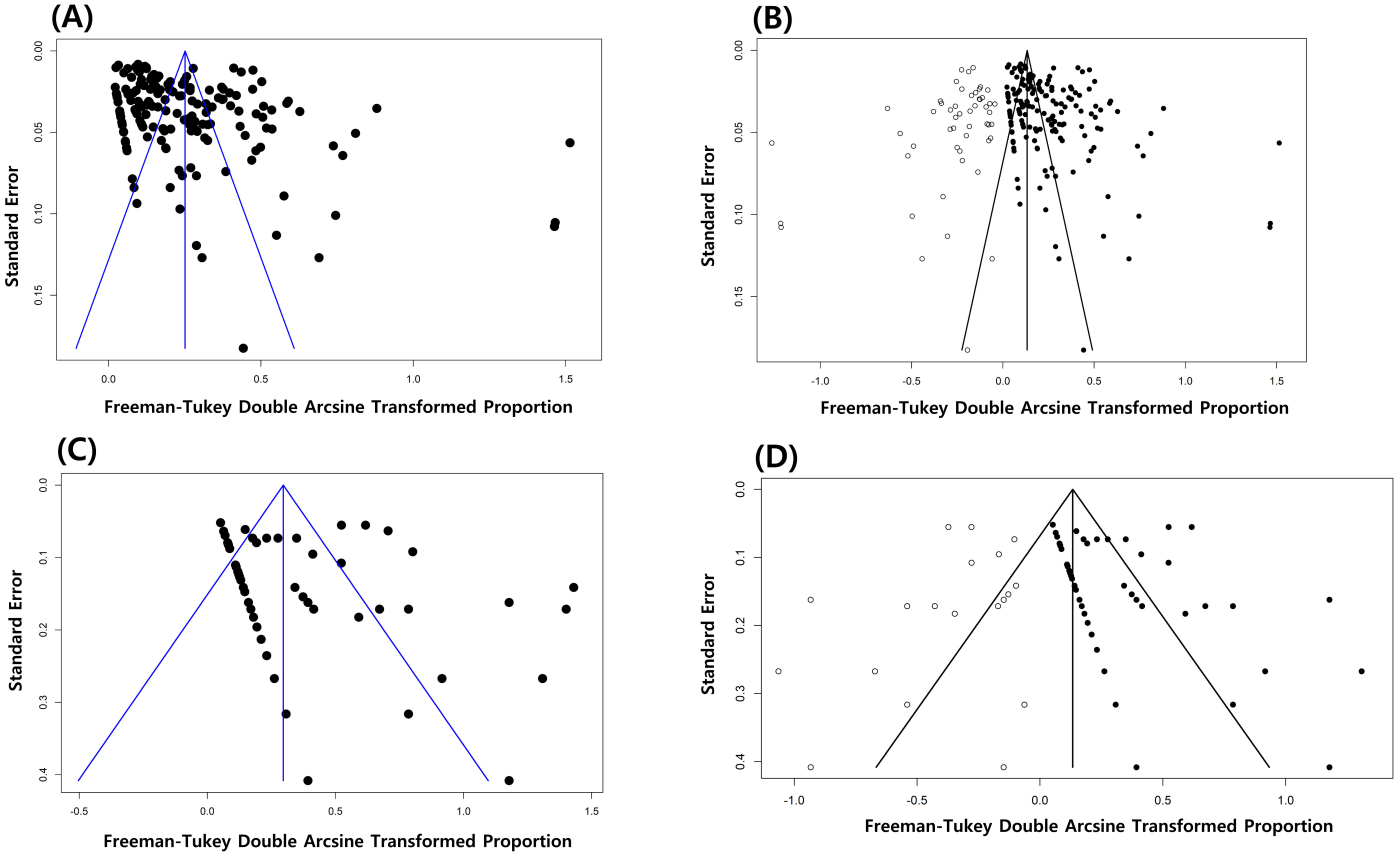
Funnel plots for publication bias on the general prevalence of *Salmonella* in wild birds **(A)**, and the corrected funnel plot including the potentially missing studies (54 studies) after applying the trim-and-fill method **(B)**. Funnel plots for publication bias on the studies investigating the resistance of *Salmonella* isolates to cephalosporins in wild birds **(C)**, and the corrected funnel plot depicting 55 likely missing studies after applying the trim-and-fill method **(D)**.

To evaluate the likelihood of publication bias, funnel plots were constructed, displaying transformed proportions on the x-axis and their corresponding standard errors on the y-axis, thereby allowing a visual assessment of the study distribution relative to their precision and estimated effect. The funnel plot for the general prevalence of *Salmonella* spp. revealed an asymmetric distribution of studies within the plot, indicating potential publication bias (Figure 7A). Further quantitative assessment was conducted using Egger's regression test, confirming the likelihood of publication bias (*t* = 4.144, *p* < 0.001). To assess the influence of publication bias on pooled prevalence, the trim-and-fill method was applied to generate a corrected estimate that accounted for potentially missing studies. The analysis identified 54 studies that were likely missing owing to publication bias (Figure 7B). Following the application of the trim-and-fill method, the prevalence estimate shifted from 5.77% (95% CI: 4.21–7.54%) to 1.28% (95% CI: 0.37–2.57%). Furthermore, the likelihood of publication bias was revealed for the cephalosporin group (Figure 7C), while Egger's regression test yielded statistically significant results (*t* = 2.773, *p* = 0.006). The trim-and-fill method indicated that 55 studies investigating resistance to cephalosporins were potentially missing (Figure 7D), changing the prevalence rate from 1.38% (95% CI: 0.04–4.06%) to 0%; this suggests that studies reporting low or no prevalence rates were unlikely to be published. The detailed results of the publication bias assessment for all antimicrobial classes are presented in Table 4.

**Table 4:** Results of the publication bias analysis in the prevalence of antimicrobial-resistant *Salmonella* spp. in wild birds.

Antimicrobial group	No. studies	Funnel plot symmetry	Egger's regression test	
			*t*-value	*p*-value^a^
Aminoglycosides	71	Symmetrical	1.589	= 0.11
Carbapenems	21	Symmetrical	0.663	= 0.51
Cephalosporins	61	Asymmetrical	2.773	= 0.006
Diaminopyrimidines	19	Symmetrical	0.665	= 0.51
Fluoroquinolones	65	Symmetrical	0.945	= 0.35
Folate pathway inhibitors	47	Symmetrical	−0.173	= 0.86
Macrolides	21	Symmetrical	0.357	= 0.72
Penicillins	67	Symmetrical	0.605	= 0.55
Penicillin/b-lactamase inhibitors	34	Symmetrical	−0.279	= 0.78
Phenicols	57	Symmetrical	1.715	= 0.092
Polymyxins	20	Symmetrical	0.83	= 0.41
Quinolones	43	Symmetrical	1.766	= 0.08
Sulfonamides	14	Symmetrical	−0.079	= 0.94
Tetracyclines	62	Symmetrical	−0.296	= 0.77

^a^ A statistically significant p-value (p < 0.05) indicates significant funnel plot asymmetry, suggesting the likelihood of publication bias.

## 4 Discussion

The global emergence and dissemination of AMR poses a substantial threat to public health and environmental sustainability, necessitating broad surveillance across diverse ecological niches. Although often overlooked, wild birds are increasingly being recognized as important factors in AMR epidemiology, functioning as environmental reservoirs, carriers of resistance genes, and potential sources of food and water contamination (Wang et al., 2017). This systematic review and meta-analysis synthesized the current evidence on the prevalence of *Salmonella* spp. and their AMR profiles in global wild bird populations, revealing distinct epidemiological patterns in the prevalence of *Salmonella* spp. among wild birds.

The current meta-analysis estimated a pooled *Salmonella* prevalence of 5.77% (95% CI: 4.21–7.54%) in wild birds (Figure 3), aligning with previous global and regional studies documenting prevalence rates varying from 0.5% to 35% (Hernandez et al., 2016; Zhou et al., 2022; Smith et al., 2023; Soltan Dallal et al., 2024). However, substantial between-study heterogeneity (*I*^2^ = 98%, *p* < 0.001) was observed potentially due to geographical, taxonomic, and methodological differences among studies. Such variability reflects the complex ecology of *Salmonella* and the diverse environments inhabited by wild birds (Smith H. G. et al., 2020; Ryan et al., 2017). Geographically, the markedly elevated prevalence reported in Poland (46.4%) and Scotland (28.16%) contrasts sharply with the complete absence of detection in Peru, New Zealand, Malaysia, and Ecuador (Table 1). Several methodological, biological, ecological, and anthropogenic factors may explain this apparent disparity. The substantially high prevalence estimate observed in Poland likely reflects localized outbreak conditions rather than background population-level prevalence. Notably, one of the two Polish studies investigating the general prevalence of *Salmonella* was conducted during a mortality event at a bird feeder site, where all sampled birds tested positive. This is consistent with well-documented patterns of *Salmonella* outbreaks occurring in high-density feeding environments, particularly urban areas, where fecal contamination facilitates rapid transmission (Hamer et al., 2012; Hernandez et al., 2012). Previous research has demonstrated that prevalence estimates of pathogens in wild birds are frequently higher in studies conducted under outbreak conditions or shortly thereafter, whereas studies based on routine, spatially distributed surveillance are more likely to reflect baseline population-level prevalence (Ntakiyisumba et al., 2023). Moreover, seasonal, ecological, and host-related factors may have further contributed to the observed prevalence as sampling was conducted during late winter, a period when birds aggregate at feeding sites due to limited food availability, thereby increasing contact rates and transmission risk (Hamer et al., 2012). Furthermore, both Polish and Scottish studies primarily targeted garden bird populations (passerine species), including greenfinches (*Carduelis chloris*), Eurasian siskins (*Carduelis spinus*), and house sparrows (*Passer domesticus*), which are known to be more susceptible to *Salmonella* infections than other avian species (Mather et al., 2016; Hernandez et al., 2012). More broadly, geographical hotspots of *Salmonella* prevalence in wild birds can be explained by an interplay between urbanization, high anthropogenic pressure (e.g. intensive agriculture, high livestock density, and environmental contamination), climate change, and wildlife-livestock interactions (Velarde et al., 2012; Allen et al., 2010; Tonelli et al., 2026). Urbanized landscapes (e.g. parks, landfills, and public feeding sites) increase birds' exposure to anthropogenic waste and human-associated strains, as shown in studies of white ibis in Florida, where *Salmonella* shedding rates increase with greater urbanization and many of the serovars match those causing human infections (Hernandez et al., 2016). Simultaneously, regions characterized by intensive livestock farming and simplified agricultural landscapes can act as focal points for *Salmonella* spillover into wild birds. High-density farms generate environmental reservoirs of *Salmonella* (via manure, runoff, and feed spillage), attract wild birds, and reduce habitat buffering, all of which raise local prevalence in bird populations near agricultural edges (Smith H. G. et al., 2020). For instance, a significantly higher *Salmonella* prevalence in wild birds near pig farms than in more natural settings was revealed in one Spanish study, indicating spillover from domestic pigs (Andrés et al., 2013). Furthermore, climatic factors such as elevated temperatures, extreme rainfall, and shifting seasonal patterns both enhance environmental survival and dispersal of *Salmonella*, thereby amplifying incidence especially in urban and agricultural regions. Surveillance data from the United States link extreme heat and rainfall with higher salmonellosis cases, particularly in counties with dense broiler operations or more urban land cover (Morgado et al., 2021), pointing toward hotspots in coastal and warm-climate agricultural zones. As such, the geographical patterns observed in the present study not only reveal the ecology and spatial distribution of *Salmonella* in wild birds, but also reflect the degree of anthropogenic influence in a region, including land-use change, agricultural intensification, urbanization, and their interconnected public health implications.

Methodological factors further influenced general prevalence estimates and complicated direct comparisons between studies. While intestinal content (50.46%) and blood samples (46.81%) yielded markedly higher prevalence estimates, these figures were derived from a significantly smaller number of studies (*n* = 1 and *n* = 2, respectively), suggesting potential overestimation or a form of selection bias toward diseased individuals. Birds that are debilitated to be captured or admitted to rehabilitation centers are more likely to be infected than healthy free-flying individuals, which may lead to inflated prevalence estimates in invasive or clinical sample types (de Souza et al., 2020; Camacho et al., 2016). In contrast, feces (6.11%) and cloacal swabs (4.79%), which were the most commonly used sample types across 62 and 69 studies, respectively, provided a more robust and representative estimate of enteric colonization and shedding in broader wild bird populations (Table 2). To further evaluate the influence of such variations, a leave-one-out sensitivity analysis was performed. This analysis identified six potential outlier studies with likely distorted or exaggerated prevalence estimates. The omission of those studies from the analysis resulted in a slight reduction of the pooled prevalence from 5.77% to 4.47%. Despite this shift, the adjusted prevalence estimate remained within a comparable range, and heterogeneity remained largely unchanged (*I*^2^ = 98% vs. 97%), indicating that the observed variability is not driven by a single outlier but rather reflects cumulative differences across studies. These findings suggest that, although certain studies may have inflated prevalence estimates due to localized or methodological factors, the overall conclusions remain robust. From a public health perspective, the adjusted prevalence estimate remains relatively high, highlighting a consistent burden of *Salmonella* in wild bird populations and underscores their potential role in environmental dissemination and zoonotic transmission. Taken together, these results emphasize the need for harmonized study designs and standardized sampling approaches to improve comparability and strengthen global surveillance efforts (Cason et al., 2025).

The incidence of *Salmonella* spp. in wild birds was significantly associated with the host taxonomic order (Table 2). Regarding bird species, the order Accipitriformes exhibited the highest prevalence of 6.85% (95% CI: 2.78–12.01%), followed by Charadriiformes (6.15%, 95% CI: 3.59–9.17%), and Pelecaniformes (5.98%, 95% CI: 0.93–13.55%), whereas Apodiformes, Gruiformes, and Anseriformes showed the lowest infection rates with 0% prevalence for each. The higher prevalence of *Salmonella* in Accipitriformes, Charadriiformes, and Pelecaniformes than in Anseriformes, Apodiformes, and Gruiformes is likely attributable to differences in ecological niches, feeding strategies, and social behavior (Plaza et al., 2019). Raptors (Accipitriformes) and piscivorous or scavenging species (Pelecaniformes, Charadriiformes) are commonly exposed to *Salmonella* through the consumption of prey, carrion, or fish derived from contaminated aquatic or anthropogenic sources (Hubálek, 2004; Smith et al., 2022b; Smith H. G. et al., 2020). For example, raptors housed in an Italian wildlife center harbored *Salmonella* strains genetically traced to contaminated raw chickens used as feed (Corradini et al., 2024). Similarly, *Salmonella enterica* has been detected in both carnivorous and aquatic wild birds in Chile, underscoring the transmission of pathogens through prey and aquatic environments (Tardone et al., 2020). In contrast, many birds in the Anseriformes, Apodiformes, and Gruiformes orders primarily utilize herbivorous or insectivorous diets, which generally present a lower risk of direct exposure to enteric pathogens. Moreover, Charadriiformes and Pelecaniformes commonly form large breeding or stopover aggregations, creating conditions conducive to fecal-oral transmission and pathogen amplification, whereas Apodiformes and Gruiformes typically exhibit dispersed foraging and social structures (Benskin et al., 2009). This underscores the importance of integrating species-specific traits, such as scavenging behavior and anthropogenic habitat use, into surveillance approaches to improve the prediction and mitigation of *Salmonella* prevalence in wild birds.

The distribution of *Salmonella* serovars in wild birds demonstrates clear epidemiological patterns, with *Salmonella enterica* serovar Typhimurium (4.12%) and *S*. Enteritidis (1.42%) consistently emerging as the most prevalent serovars across wildlife surveillance studies (Table 2). This dominance reveals their broad host range and ecological adaptability, which enable these serovars to infect multiple vertebrate hosts, including wild birds, livestock, and humans. *S*. Typhimurium, in particular, is recognized as a serovar capable of circulating across diverse animal reservoirs, including poultry, cattle, pigs, and wild birds, thereby facilitating cross-species transmission and increasing opportunities for environmental dissemination (Bajwa et al., 2026; Lamichhane et al., 2024). Several studies have also reported that *S. typhimurium* is the most common serotype isolated during salmonellosis outbreaks in wild songbirds, occasionally leading to large mortality events and highlighting its strong ecological association with avian hosts (Patel et al., 2023). Similarly, *S*. Enteritidis is strongly linked to avian reservoirs, particularly poultry and egg-producing birds, and remains one of the leading causes of foodborne salmonellosis in humans worldwide, largely due to contamination of eggs and poultry products (Alegria-Moran et al., 2017; Zhou et al., 2026; Hald et al., 2012). The prominence of these serovars in wild bird populations therefore has important public health implications, as they overlap with the dominant serovars responsible for human infections and livestock outbreaks globally. Indeed, the circulation of *S. typhimurium* and *S. enteritidis* across wildlife, livestock, and human populations highlights the interconnected epidemiology of salmonellosis within the wildlife-livestock-human interface, where wild birds may contribute to the environmental dissemination and long-distance spread of zoonotic strains (Bajwa et al., 2026). Although other serovars such as *S. virchow, S. infantis*, and region-specific serotypes are occasionally detected in wildlife, their lower prevalence suggests a more limited ecological distribution compared with these globally dominant serovars. Overall, these findings reinforce the importance of monitoring serovar diversity in wild birds within a One Health surveillance framework, as the persistence of zoonotic serovars in wildlife reservoirs may facilitate the emergence, maintenance, and geographic dissemination of strains that are epidemiologically relevant to both livestock production systems and human health.

Despite an increasing number of publications over the past 25-year span (from 2000 to 2025), the lack of any statistically significant temporal trend in prevalence indicates that the overall prevalence of *Salmonella* spp. in wild birds has remained relatively steady. This finding of temporal stability is supported by several regional and global studies that have similarly failed to detect any significant increase in *Salmonella* prevalence in wild birds over time, indicating a state of equilibrium in host-pathogen dynamics within these populations (Hernandez et al., 2012). This equilibrium may be maintained by an intricate balance of several factors, including the constant introduction of *Salmonella* from environmental and anthropogenic sources, and natural resistance and immunity in wild birds (Mughini-Gras et al., 2018). However, some conflicting findings have been documented in previous regional investigations that reported rising trends in pathogen prevalence associated with intensified poultry production and agricultural expansion. For example, Minias (2020) recognized divergent temporal trends in the occurrence of *Salmonella* in birds, while (Hall and Saito, 2008) reported significant temporal relationships between *Salmonella* occurrence and avian wildlife mortality (Minias, 2020; Hall and Saito, 2008). Nonetheless, the occurrence of localized outbreaks and interannual fluctuations underscores the need for further sustained surveillance, particularly in regions undergoing rapid agricultural expansion and environmental transformation. Collectively, these findings emphasize that the epidemiological dynamics of *Salmonella* in wild birds are less affected by global temporal trends than by regional ecological and anthropogenic factors. Consequently, although the global average prevalence remains steady, the threat of sporadic emergence necessitates targeted and ongoing monitoring of high-risk regions.

The AMR landscape of *Salmonella* isolates from wild birds was characterized by substantial heterogeneity, encompassing both very low resistance and moderate to high resistance to medically important antimicrobials including those belonging to CIAs group. While overall resistance to CIAs such as fluoroquinolones (0.37%) and carbapenems (0.39%) remained very low, indicating that these therapeutic options largely retain their effectiveness against *Salmonella*, higher resistance rates were detected for other antimicrobial classes, particularly macrolides (29.64%), monobactams (22.89%), and penicillins (14.71%; Table 3). The resistance to these antimicrobial classes is typically acquired and mediated by mobile genetic elements or chromosomal mutations, including macrolide-modifying enzymes (e.g., *mph* genes), efflux pumps such as MacAB, and β-lactamase enzymes conferring resistance to β-lactam antibiotics, thereby reflecting environmental exposure to antimicrobial residues or resistant bacteria of anthropogenic origin (Chaudhari et al., 2023). In contrast, the markedly high resistance rates observed for lincosamides (93.70%) and glycopeptides (91.98%) primarily reflect the intrinsic resistance of *Salmonella* and other Gram-negative bacteria (Aqib and Alsayeqh, 2022). This intrinsic resistance is largely attributable to structural characteristics such as the outer membrane permeability barrier and associated transport systems that prevent these antimicrobial agents from reaching their cellular targets (Vestö et al., 2019). Consequently, resistance to glycopeptides and lincosamides should not be interpreted as evidence of acquired antimicrobial resistance or environmental selection pressure. Overall, the detected resistance to medically relevant antimicrobial classes suggests that wild birds may serve not only as reservoirs of naturally resistant *Salmonella* strains but also as environmental sentinels of anthropogenically driven resistance dynamics.

Univariate meta-regression analysis identified a significant association between publication year and AMR prevalence for penicillins with β-lactamase inhibitors (*R*^2^ = 66.75%), fluoroquinolones (*R*^2^ = 19.21%), aminoglycosides (*R*^2^ = 11.05%), and macrolides (*R*^2^ = 4.60%), indicating a temporal increase in AMR in wild birds (Table 3). Temporal increases in antimicrobial resistance are widely recognized as indicators of sustained antimicrobial exposure and selective processes acting on bacterial populations in environmental reservoirs (Fletcher, 2015; Wang et al., 2023). Similar time-based surges in resistance have been reported in Europe, where growing resistance to aminoglycosides and β-lactamase inhibitors in wild birds has been associated with the dissemination of resistance genes such as *blaCTX-M* and *aac(3)-IIa* (Guenther et al., 2011; Atterby et al., 2016). Collectively, these findings indicate considerable evolution over time, likely shaped by changes in antimicrobial usage and environmental selective pressures (Raban et al., 2021; Smit et al., 2025). The subgroup analysis further indicated that geographical region had a significant influence on AMR prevalence in wild birds for most antimicrobial groups. For example, alarming rates have been reported in Trinidad and Tobago (100% resistance to cephalosporins), Argentina and Brazil (100% resistance to macrolides), Bulgaria (100% resistance to penicillins), Egypt (83.3% resistance to quinolones), Poland (94.4% resistance to sulfonamides), and Italy (100% resistance to sulfonamides). Conversely, minimal or absent resistance in countries such as Switzerland, Singapore, and Portugal highlight the potential for effective AMR control strategies and lower exposure to resistant bacteria (Figure 4). These findings further reflect regional differences in antimicrobial usage in the human and veterinary sectors and/or varying levels of environmental contamination from human and livestock waste (Van Boeckel et al., 2015; Nashwan et al., 2024). Additionally, differences in national policy frameworks regarding antibiotic use in agriculture and medicine play a key role, as countries with stricter regulations and stronger antimicrobial stewardship programs likely exert lower environmental selective pressure, thereby reducing the amplification and spillover of resistant pathogens into wildlife populations (Van Boeckel et al., 2015).

Subgroup analysis indicated that the AST method did not significantly influence the pooled AMR prevalence estimates across antimicrobial classes, as no statistically significant differences were observed between dilution methods, disk diffusion, and automated systems (*p* > 0.05). These findings suggest that methodological variation in susceptibility testing was not a major contributor to heterogeneity in AMR prevalence among *Salmonella* isolates in wild bird. However, the lack of statistical significance should be interpreted cautiously, as it may partly reflect the unequal distribution of testing approaches across the included studies. Specifically, most studies relied on the disk diffusion method (*n* = 52), while fewer used dilution-based techniques such as broth microdilution or agar dilution (*n* = 17), and only two studies employed automated systems (VITEK). Such imbalance may reduce the statistical power of subgroup comparisons and limit the ability to detect methodological differences when they exist. From a methodological perspective, dilution methods that determine the minimum inhibitory concentration (MIC) are widely considered the reference standard for AST, whereas disk diffusion remains the most commonly used approach in routine laboratories because of its simplicity and lower cost (Westrell, 2016). The predominance of disk diffusion in the included studies likely reflects common laboratory practice in wildlife microbiology, but future surveillance efforts would benefit from increased use of MIC-based methods or standardized automated systems, which could improve comparability across studies and enhance the precision of AMR monitoring in wildlife reservoirs.

Another critical finding of this study was the significant differences in AMR rates across continents, especially for aminoglycosides, cephalosporins, diaminopyrimidines, and quinolones. Notably, the highest resistance rates were detected in Africa, whereas Oceania and North America exhibited the lowest resistance. The high AMR prevalence rates in Africa could further be attributed to several interrelated drivers. Environmental contamination from untreated sewage, slaughterhouse effluents, agricultural runoff, and poorly managed landfills is a significant source of resistant bacteria, particularly in regions with limited wastewater treatment infrastructure (Manyi-Loh et al., 2018). In addition, the widespread and often unregulated use of antimicrobials in both livestock production and human medicine intensifies selection pressure, facilitating the dissemination of resistance determinants into the environment (Salam et al., 2023; Ahmed et al., 2024). Furthermore, close ecological interactions between wild birds, domestic animals, and humans, particularly in peri-urban and rural settings such as open markets, shared water sources, and carcass disposal sites, further amplify the opportunities for bacterial exchange (Naguib et al., 2021). In contrast, a lower AMR prevalence in wild birds is generally observed in North America and Oceania, where robust antibiotic stewardship policies, widespread wastewater treatment, stricter veterinary regulations, and higher levels of farm biosecurity collectively reduce environmental reservoirs of resistant bacteria (Hendriksen et al., 2019). It is also important to note that methodological differences may influence these observed continental gradients, as many African studies focus on high-risk sampling sites, such as abattoirs and landfills, whereas surveillance in North America and Oceania often encompasses broader, routine sampling in less contaminated environments, leading to lower prevalence estimates (Mbuthia and Hoza, 2025). Furthermore, the higher prevalence of AMR reported in Africa should be interpreted with caution, given that this estimate was derived from a single study included in the meta-analysis. Consequently, these findings are unlikely to represent the continent's diverse wild bird populations. Further region-specific investigations are necessary to draw robust conclusions regarding AMR rates in wild birds across Africa.

This study has several limitations that need to be acknowledged. First, the prevalence rates among the individual studies showed substantial heterogeneity. Although we used a couple of variables in the subgroup analyses to explore the sources of heterogeneity, other important factors, such as detected *Salmonella* serovars, seasonality, and precise environmental factors, were not investigated in the current study, as they were often not reported in the primary studies included in the meta-analysis. It is also important to mention that the findings of the meta-analysis indicated a likely presence of publication bias, which to some extent could have affected confidence in the pooled prevalence estimate. After applying the trim-and-fill method, the pooled prevalence decreased from 5.77% (95% CI: 4.21–7.54%) to 1.28% (95% CI: 0.37–2.57%), suggesting that small studies reporting zero or low prevalence were likely missing, consistent with the well-recognized “file drawer problem,” whereby studies with non-significant or negative findings are less likely to be published (Lishner, 2022). However, the presence of publication bias should be interpreted with caution. The primary studies included in this meta-analysis are all observational, cross-sectional studies, and thus do not calculate significant levels for their results. The tools designed to assess the publication bias are mainly for randomized controlled trials and they follow the assumption that the study publication is influenced by its statistical significance which is not the case for observational studies (Wang, 2017; Maulik et al., 2011). Furthermore, these approaches assume that dispersion of effect sizes is driven by sampling error and do not fully account for the possibility that studies may estimate different true effects (Harrer et al., 2021). Given the significant heterogeneity observed among the included studies, it is also likely that funnel plot asymmetry was caused by between-study heterogeneity rather than true publication bias. According to Peters et al. (2007), when between-study heterogeneity exists, the trim and fill method is generally recommended as a form of sensitivity analysis rather than a definitive estimate of the pooled effect (Peters et al., 2007). Therefore, the adjusted estimate (1.28%) should be regarded primarily as a sensitivity analysis representing a conservative lower-bound scenario rather than a definitive estimate of global *Salmonella* prevalence in wild bird populations.

Another limitation of this meta-analysis is the uneven geographical distribution of the available studies. Only a small number of studies were conducted in certain regions, particularly Africa (*n* = 4) and Oceania (*n* = 6). Consequently, the prevalence and AMR estimates derived for these continents should be interpreted with caution, as they may not adequately represent the true epidemiological situation of *Salmonella* in wild bird populations in these regions. The limited number of studies may reduce the precision and generalizability of the pooled estimates and may reflect gaps in wildlife surveillance efforts. Therefore, additional well-designed epidemiological studies are needed to generate more robust and regionally representative data on the prevalence and AMR patterns of *Salmonella* in wild bird populations from these continents. Finally, data for certain antimicrobial groups, including glycopeptides, glycylcyclines, lincosamides, monobactams, phosphonics, and polypeptides, were sparse, making subgroup and meta-regression analyses unsuitable. Despite these limitations, the findings of this study provide a more robust estimate of *Salmonella* spp. prevalence and AMR status in global wild bird populations than those obtained from a single study.

## 5 Conclusion

This investigation highlights that wild birds are important reservoirs and disseminators of *Salmonella* serovars, many of which are resistant to multiple antimicrobial agents, including those classified as critically important in human and veterinary medicine. From a public health perspective, four key conclusions can be drawn from this study. First, significant variation in *Salmonella* prevalence across avian taxonomic orders has been revealed, with Accipitriformes, Charadriiformes, and Pelecaniformes exhibiting the highest infection rates, while several other orders showed no detectable prevalence. This pattern suggests that ecological and behavioral traits associated with certain bird groups, such as predatory feeding behavior in raptors or the frequent contact of shorebirds and waterbirds with contaminated aquatic environments, may influence exposure to *Salmonella* and contribute to the observed differences in prevalence among wild bird taxa. These findings suggest that raptors and aquatic birds may serve as valuable sentinel species for monitoring environmental circulation of *Salmonella* and could therefore represent priority targets for future wildlife surveillance and One Health monitoring programs. Second, the predominance of *S. typhimurium* and *S. enteritidis* in wild birds indicates that wildlife reservoirs may contribute to the environmental persistence and dissemination of the same *Salmonella* serovars most frequently associated with human and livestock infections. Third, the significant temporal increase in resistance to fluoroquinolones, penicillin/β-lactamase inhibitors, aminoglycosides, and macrolides provides strong evidence of increasing environmental selective pressure shaping antimicrobial resistance in wildlife-associated *Salmonella*. The steady rise in resistance to these CIAs classes likely reflects sustained environmental exposure to antimicrobial residues and resistant bacteria originating from anthropogenic sources. Regional and global agricultural intensification, land-use change, and urban expansion further heighten opportunities for pathogen transmission at the human-livestock-wildlife interface. These findings underscore the role of environmental reservoirs as dynamic interfaces for resistance evolution and dissemination and highlight the importance of long-term ecological surveillance within a One Health framework to mitigate emerging public health risks. Fourth, the high prevalence of AMR *Salmonella* spp. in wild birds across all continents, illustrates the important role these avian species may play in the global dissemination of resistance. The migratory behavior of many species, together with their diverse feeding strategies and ability to exploit degraded habitats, facilitates the transboundary spread of resistant bacteria and associated resistance determinants. Climate change may further add another layer of complexity by altering migration routes, host distributions, and environmental pathogen persistence, thereby affecting migration patterns and cross-species interactions.

Overall, these insights emphasize the need for integrated and internationally coordinated surveillance frameworks that account not only for pathogen prevalence and AMR profiles, but also for the ecological and environmental drivers governing their distribution. Strengthening antibiotic stewardship, improving waste and environmental management, and safeguarding natural habitats are essential strategies to mitigate the escalating public health risks associated with *Salmonella* in wild bird populations.

## Data Availability

The original contributions presented in the study are included in the article/supplementary material, further inquiries can be directed to the corresponding author.

## Supplementary material

Supplementary File S1 The PRISMA 2020 guidelines check list

Supplementary File S2 Characteristics of studies included in the meta-analysis of the global prevalence of *Salmonella* spp. in wild birds.

Supplementary File S3 Characteristics of studies included in the meta-analysis of the prevalence of antimicrobial-resistant *Salmonella* spp. in wild bird populations.

Supplementary File S4 Sensitivity analysis of the prevalence of *Salmonella* in wild bird populations. the prevalence estimates (right) and their corresponding 95% confidence intervals were computed by leaving out each study (left).

Supplementary File S5 Detailed subgroup analysis results for AMR *Salmonella* serovar prevalence, including 95% confidence intervals by continent, AST method, and sample source.
