# Supplementary figures and images for: Wild birds as environmental reservoirs of antimicrobial-resistant Salmonella: a global systematic review and meta-analysis
Source: Front Microbiol. 2026 Apr 24;17:1787396. doi: 10.3389/fmicb.2026.1787396 (PMC13152869; doi:10.3389/fmicb.2026.1787396)

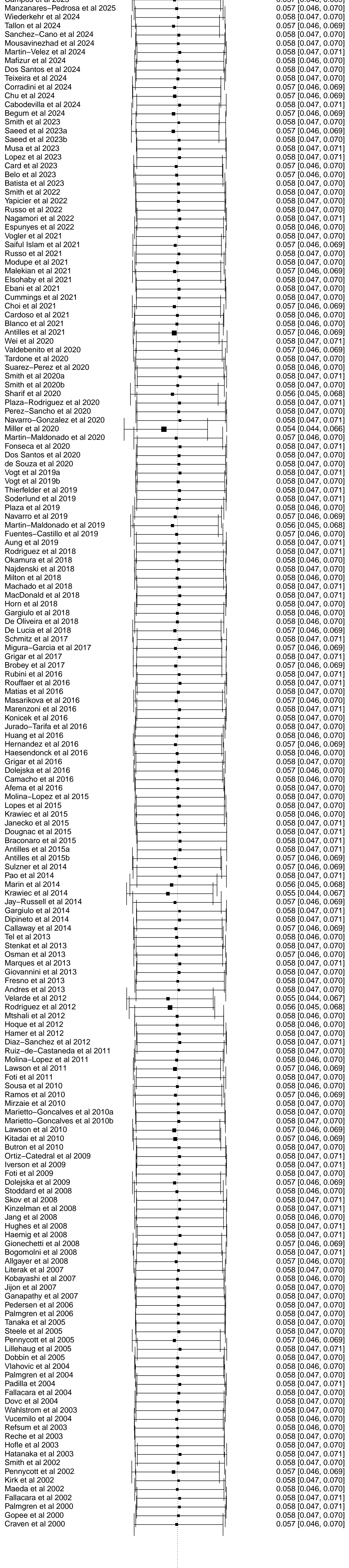

0.040 0.050 0.060 0.070  
Salmonella prevalence leaving out each study

Supplement: Supplementary File S5 — Detailed subgroup analysis results for AMR Salmonella serovar prevalence, including 95% confidence intervals by continent, AST method, and sample source. [file Presentation_1.PDF]
